# Simple and reliable method for predicting extracorporeal membrane oxygenation flow rates and circuit pressures

**DOI:** 10.1186/s40635-026-00870-z

**Published:** 2026-03-04

**Authors:** Kazuhiro Takahashi, Seiga Takahashi, Yusuke Takei, Yu Kaiho, Takahiro Imaizumi, Kenji Kikuchi, Takuji Ishikawa, Yutaka Ejima, Masanori Yamauchi

**Affiliations:** 1 Department of Anesthesiology and Perioperative Medicine, Tohoku University Graduate School of Medicine, 1-1 Seiryo-Machi, Aoba-Ku, Sendai, Miyagi 980-8574 Japan; 2 Division of Clinical Engineering, Clinical Technology, Tohoku University Hospital, Sendai, Japan; 3 Department of Clinical Research Education, Nagoya University Graduate School of Medicine, Nagoya, Japan; 4 Department of Finemechanics, Graduate School of Engineering, Tohoku University, Sendai, Japan; 5 Graduate School of Biomedical Engineering, Tohoku University, Sendai, Japan; 6 Division of Surgical Center and Supply, Sterilization, Tohoku University Hospital, Sendai, Japan

**Keywords:** Extracorporeal membrane oxygenation, Fluid dynamics, Cannulation, Venovenous ECMO, Extracorporeal circulation

## Abstract

**Background:**

Venovenous extracorporeal membrane oxygenation (ECMO) is essential for patients with severe respiratory failure who do not respond to conventional mechanical ventilation. Adequate ECMO flow and safe circuit pressure are critical; however, cannula selection, which has a great impact on these factors, is often based on empirical judgment. This study aimed to develop a simple predictive method based on fluid dynamics for estimating ECMO flow rate and circuit pressures (P1: pre-pump, P2: pre-oxygenator, and P3: post-oxygenator). This experimental predictive model study compared the calculated and measured ECMO parameters across 36 combinations of cannula sizes, pump speeds, and bed heights. A laboratory-based ECMO circuit model was assembled with various drainage and return cannulas, an oxygenator, tubing, and a centrifugal pump. The circuit was primed with a 33% glycerin solution and tested across the 36 combinations. A four-step prediction method was applied: (1) modeling the pressure–flow relationships of ECMO components and the pump using manufacturer data; (2) identifying the expected flow rate by locating the intersection of the total circuit resistance and pump output curves; (3) sequentially calculating pressure drops across the circuit; and (4) adjusting pressures based on bed height.

**Results:**

The predicted flow rate and circuit pressure values were compared to experimental measurements across the 36 combinations. The calculated and measured values showed strong agreement (R^2^ = 0.96–0.97), and predictions were significant. Notably, bed height alterations were confirmed to affect circuit pressure but not flow rate.

**Conclusions:**

Our newly developed method reliably predicts the ECMO flow rate and circuit pressure. Hence, it can be considered a valuable tool for preemptively selecting the optimal cannula size for ECMO, thus improving patient safety and circuit management. Furthermore, it may be a valuable educational tool, making complex hemodynamic concepts more intuitive for trainees.

**Supplementary Information:**

The online version contains supplementary material available at 10.1186/s40635-026-00870-z.

## Background

Venovenous extracorporeal membrane oxygenation (VV-ECMO) is a life-saving intervention for patients with severe respiratory failure who do not respond to conventional mechanical ventilation. By providing gas exchange support, VV-ECMO allows the lungs to rest and recover [1].

To achieve effective ECMO flow while preventing complications such as air entrainment and hemolysis, circuit pressures must be maintained within a safe range. Several studies have investigated the effects of cannula size on the ECMO flow and circuit pressures. The Extracorporeal Life Support Organization guideline describes a 25-Fr drainage cannula as an example capable of maintaining an appropriate ECMO flow rate in a 180-cm-tall patient [2]. Robak et al. (2022) also presented data from multiple clinical cases showing the ECMO flow, circuit pressures, and corresponding pump rotational speeds when 25- and 23-Fr drainage cannulas were used [3]. Lehle et al. (2014), using clinical data and linear regression analysis, demonstrated that the pressure drop across the drainage cannula can be estimated based on cannula size and ECMO flow rate [4].

However, no standardized or validated method exists to predict the optimal cannula size required to maintain adequate ECMO flow and circuit pressures. Consequently, cannula selection is usually guided by institutional experience or empirical judgment. Selecting the appropriate size becomes particularly challenging when a patient’s vessel diameter is relatively small and an unfamiliar cannula must be used.

A VV-ECMO circuit is mainly composed of drainage and return cannulas, oxygenators (gas blenders and heat exchangers), and pumps. Each manufacturer publishes their own pressure–flow characteristics for these components, and thus, they vary. Importantly, the larger the cannula diameter, the lower the pressure required to achieve the target flow rate. This pressure–flow relationship of the cannula can be described using a simple quadratic curve [5]. Similarly, greater pressure is required to allow higher blood flow through the oxygenator [6]. Higher centrifugal pump rotational speeds yield higher ECMO flow rates [7]. However, although studies have characterized the individual components in detail, the combined impact of these components on ECMO flow rate and circuit pressure remains unknown.

Therefore, this study aimed to devise a method to calculate ECMO flow rate and circuit pressures (pre-pump pressure P1, pre-oxygenator pressure P2, and post-oxygenator pressure P3) based on the characteristics of each component. This novel method is grounded in fluid dynamics principles. Particularly, the method is based on the following principles: (1) ECMO circuit components consume pressure; (2) the pump supplies pressure; and (3) the pressure consumed equals the pressure supplied.

## Methods

### Study design

The ECMO flow rate and circuit pressures calculated using the following methods were compared with experimental data to evaluate their validity. In addition, three patients were evaluated to further assess the accuracy of the proposed formulas (Appendix 1 in Additional File 1). All three patients were supported using the same ECMO circuit as in the in vitro study. Two patients underwent VV-ECMO for lung transplantation, and one patient for tracheal stent placement. In all cases, cannulation was performed in the operating room, and VV-ECMO was discontinued in the operating room after several hours of surgery. The need for ethical approval for this study was waived by the Ethics Committee of Tohoku University Graduate School of Medicine because approval for case reports is not required as per the Ethical Guidelines for Medical and Biological Research Involving Human Subjects in Japan. Individual consent was obtained from all the patients.

### Method for predicting ECMO flow rate and P1, P2, and P3

The equation to predict ECMO flow rate and circuit pressures comprised four steps (Table S1 in Additional File 1). The first two steps involved calculating the ECMO flow rate, while the remaining two steps involved calculating circuit pressures.

Step 1: creating an approximate curve for each part of the ECMO and the pump.

**Fig. 1:**
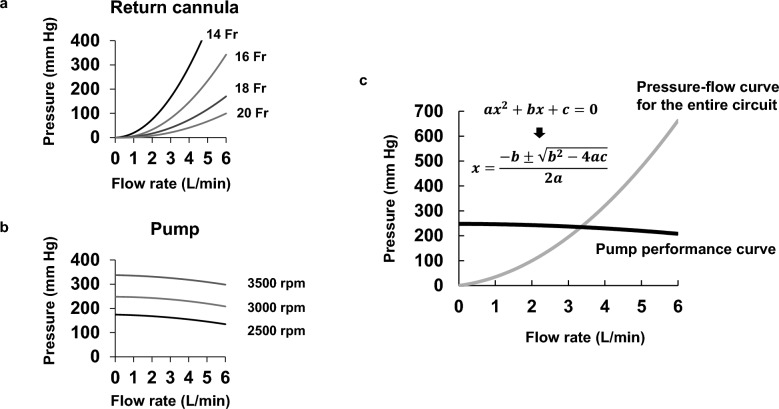
Pressure–flow relationships for extracorporeal membrane oxygenation components. **a** Approximate pressure–flow curves for the return cannula: The horizontal axis indicates the flow rate through the cannula, and the vertical axis shows the pressure drop across the cannula. A smaller cannula’s inner diameter results in a steeper curve. **b** Pump performance curves: the horizontal axis represents the flow rate through the pump, and the vertical axis represents the pressure generated by the pump. Higher pump speeds shift the curve upward. **c** Intersection of the circuit pressure–flow curve and the pump performance curve: the entire circuit’s pressure–flow and the pump’s performance curve are superimposed. The horizontal axis indicates the flow rate, and the vertical axis indicates pressure. The intersection of the two curves represents the operating point, and its x-coordinate corresponds to the predicted extracorporeal membrane oxygenation flow rate

Approximate curves (P = (K1) * Q^2 + (K2) * Q + K3) were generated by extracting multiple data points (flow–pressure pairs) from product catalogs and fitting quadratic approximation curves to these points. P denotes pressure (mmHg), Q denotes flow rate (L/min), and K represents a coefficient unique to each cannula or pump. Regarding the approximation curve of the cannula, K1 in the quadratic curve reflects the density of the fluid, while K2 reflects the viscosity of the fluid [5]. Regarding the approximation curve of the pump, K3 reflects “Shut-off head”, that is, the maximum pressure developed by a pump when the circuit is obstructed and no flow passes through the system. Figure 1a and 1b shows the approximate pressure–flow curves of the return cannula and the performance curves of the pump used. Table S2 (Additional File 1) lists the approximation curve coefficients used in the study.

Step 2: finding the intersection of the approximate curves of the circuit and the pump.

The pressure–flow curves of the individual parts were added together to calculate the pressure–flow curve for the entire circuit (Figure S1 in Additional File 1). The intersection of this pressure–flow curve and the pump performance curve was then determined. As both curves were expressed as quadratic functions, the intersection could be calculated using the quadratic formula (Fig. 1c).

Step 3: calculating the pressure within the circuit from the drainage side.

**Fig. 2:**
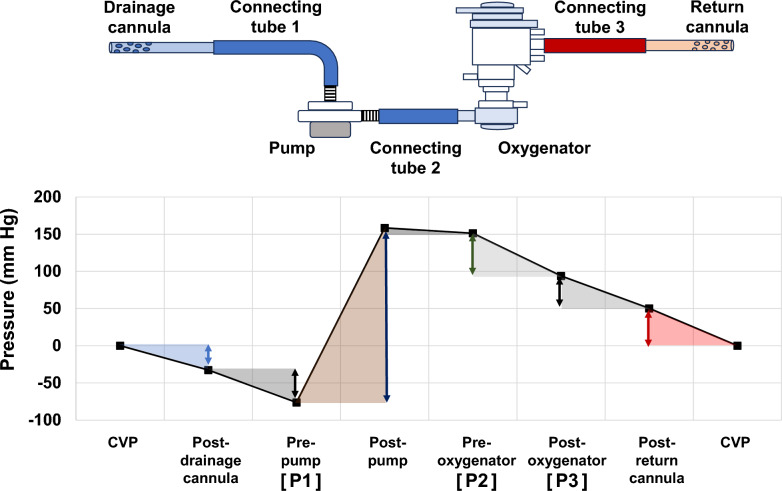
Sequential calculation of circuit pressures from drainage to return. Circuit pressures (P1, P2, and P3) are calculated by sequentially subtracting or adding pressure drops or gains from the CVP. For example, subtracting the pressure loss of the drainage cannula and connection part 1 from the CVP, adding the pump pressure, and subtracting the pressure loss of connection part 2 yields P2. P1; pre-pump pressure; P2, pre-oxygenator pressure; P3, post-oxygenator pressure; CVP; central venous pressure

The pressure change occurring in each component was calculated using the approximate pressure–flow curves (step 1) and ECMO flow rate (step 2). Subsequently, by sequentially adding and subtracting these pressure changes from the drainage side, all circuit pressures, including P1, P2, and P3, were obtained. For example, the pre-oxygenator pressure (P2) was calculated by subtracting the pressure drops of the drainage cannula and connecting tube 1 from the central venous pressure, adding the pump’s pressure supply, and then subtracting the pressure drop of connecting tube 2 (Fig. 2).

Step 4: adjusting for bed height.

As the final step, circuit pressures were adjusted according to bed height. The laws of fluid mechanics (extended Bernoulli’s equation) indicate that the potential and pressure energies of the fluid in the circuit are interchangeable (Appendix 2 in Additional File 1). By elevating the bed, the potential energy of the relatively lower circuit section, which includes the pump, connection tube 2, and oxygenator, decreases, whereas the pressure energy (P1, P2, and P3) increases (Figure S2 in Additional File 1). If the bed height was increased by 40 cm, a pressure of 40 cmH_2_O/1.36 = 29.4 mm Hg was added to the circuit pressure. This was because the specific gravity of blood is approximately 1.05; its effect was minimal and was omitted from the equation to simplify calculations.

### Experimental procedure

**Fig. 3:**
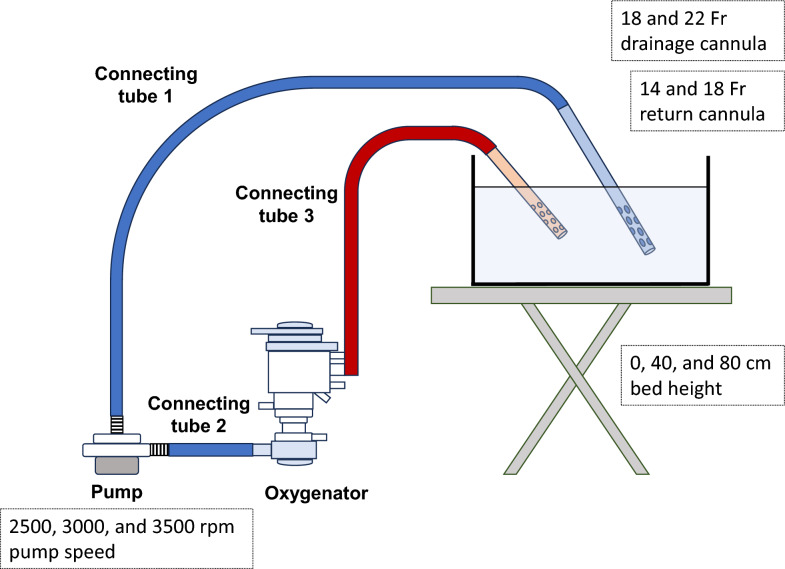
Experimental model setup. The drainage cannula, connecting tube 1, pump, connecting tube 2, oxygenator, connecting tube 3, and return cannula are assembled in series and primed with a 33% w/v glycerin solution. Both drainage and return cannulas are immersed in the same glycerin solution

The experimental model is shown in Fig. 3. The ECMO components, including the drainage cannula, connecting tube 1, pump, connecting tube 2, oxygenator, connecting tube 3, and return cannula (Senko Medical Instrument Manufacturing Co., Tokyo, Japan), were assembled in series and primed with a 33% W glycerin solution. Both the drainage and return cannulas were submerged in the same 33% W glycerin bath solution. Glycerin solutions behave as Newtonian fluids and therefore do not exhibit non-Newtonian properties such as the shear-thinning behavior observed in blood [8]. Despite this difference, they are widely employed as blood analogs [9], because their viscosity can be tuned easily to approximate that of whole blood. In the present study, a 33% glycerin solution was used to reproduce the testing conditions adopted by the cannula manufacturer, Senko medical instrument. At 20 °C, the kinematic viscosity of this solution is approximately 2.7 mm^2^/s [10], which is comparable to that of blood with a hematocrit of approximately 30% [9].

Pump speeds were set to 2,500, 3,000, and 3,500 rpm. ECMO flow rates and pressures (P1, P2, and P3) were recorded under stable conditions. A total of 36 combinations (2 sizes each of drainage and return cannulas, 3 pump speeds, and 3 bed heights) were evaluated (Fig. 3).

### Statistical analysis

Flow rates and pressures (P1, P2, and P3) predicted by the computational model were compared with experimental measurements. Bias was defined as the difference between computational and experimental values. Relative error (%) was defined as the difference between the measured and predicted values divided by the measured value, multiplied by 100. The coefficient of determination (R^2^) and root mean square deviation (RMSD) were used to evaluate the agreement between the predictions from the four-step model and the experimental data. Detailed statistical analyses are provided in Appendix 3 (Additional File 1). All statistical analyses were conducted using R version 4.5.1 (The R Foundation for Statistical Computing, Vienna, Austria, 2025–6-13) and Microsoft Excel (Microsoft Corp., Redmond, WA, USA, 2021).

## Results

### Effect of cannula size, pump speed, and bed height on ECMO flow rate

**Fig. 4:**
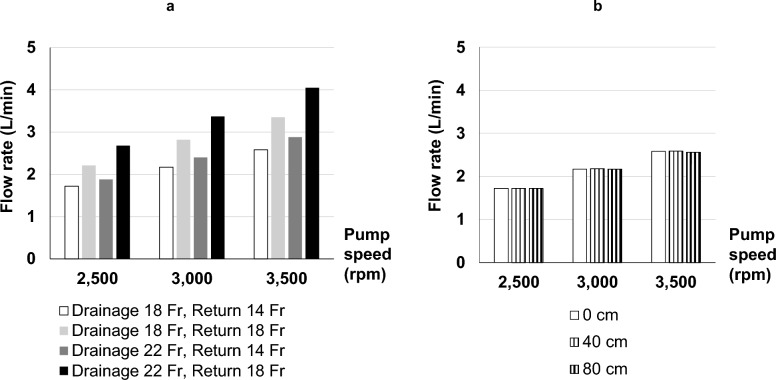
Effect of cannula size and bed height on ECMO flow rate. **a** Bar graph showing that increasing the diameter of either the return or drainage cannula increases the ECMO flow rate. The white, light gray, dark gray, and black bar graphs represent drainage and return cannula of 18 Fr and 14 Fr, 18 Fr and 18 Fr, 22 Fr and 14 Fr, and 22 Fr and 18 Fr, respectively. The vertical axis shows the experimental values of the ECMO flow rate, and the horizontal axis indicates the pump rotation speed. **b** Bar graph showing that bed height does not affect ECMO flow rate. The white, narrow striped, and wide striped bar graphs represent bed heights of 0, 40, and 80 cm, respectively. The vertical axis shows the experimental values of the flow rate, and the horizontal axis indicates the pump rotation speed. ECMO; extracorporeal membrane oxygenation

Figure 4 illustrates the effects of cannula size and bed height on the ECMO flow rate at each pump speed. Increasing the return cannula diameter from 14 to 18 Fr resulted in a greater increase in ECMO flow rate compared with an increase in the drainage cannula diameter from 18 to 22 Fr. A similar trend was observed at higher pump speeds. Conversely, bed height did not affect the ECMO flow rate.

### Effect of cannula size, pump speed, and bed height on circuit pressure in the experiment

**Fig. 5:**
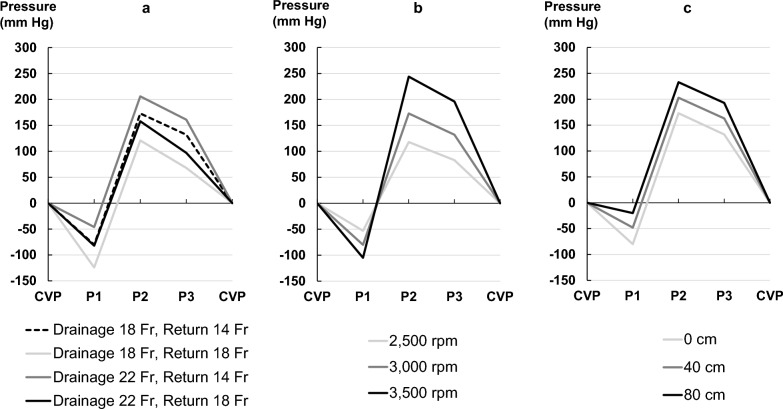
Effects of cannula size, pump speed, and bed height on circuit pressures. **a** Line graph: under a combination of pump speed of 3000 rpm and bed height of 0 cm, increasing the return cannula diameter reduces P1, P2, and P3; increasing the drainage cannula diameter increases P1, P2, and P3. The dashed line and light gray, dark gray, and black line graphs represent drainage and return cannula of 18 Fr and 14 Fr, 18 Fr and 18 Fr, 22 Fr and 14 Fr, and 22 Fr and 18 Fr, respectively. **b** Line graph: under a combination of drainage and return cannulas of 18 Fr and 14 Fr and bed height of 0 cm, higher pump speed decreases P1 but increases P2 and P3. The light gray, dark gray, and black line graphs represent pump speeds of 2,500; 3,000; and 3,500 rpm, respectively. **c** Line graph: under a combination of drainage and return cannula of 18 Fr and 14 Fr, respectively, and pump speed of 3000 rpm, greater bed height increases P1, P2, and P3. The light gray, dark gray, and black line graphs represent bed heights of 0, 40, and 80 cm, respectively. P1; pre-pump pressure, P2; pre-oxygenator pressure, and P3; post-oxygenator pressure

Figure 5 shows the effects of cannula size, pump speed, and bed height on P1, P2, and P3. As shown in Fig. 5a, P1, P2, and P3 all increased when the drainage cannula size was increased from 18 to 22 Fr while the return cannula size remained unchanged (14 Fr). In contrast, P1, P2, and P3 decreased when the return cannula size increased from 14 to 18 Fr while the drainage cannula remained unchanged (18 Fr). An increased pump speed resulted in decreased P1, whereas P2 and P3 were increased (Fig. 5b). Increasing bed height also led to increases in P1, P2, and P3 (Fig. 5c).

### Predictive performance of the equation

**Fig. 6:**
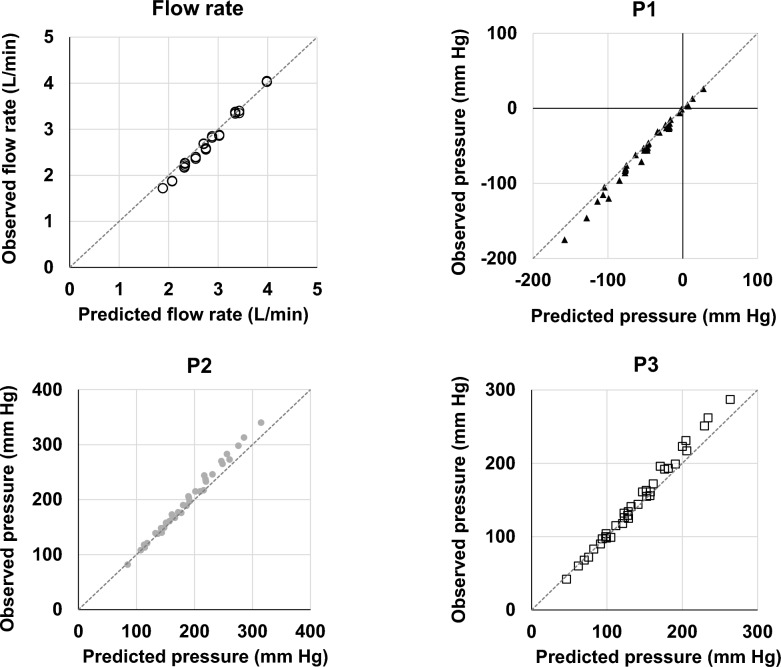
Observed versus predicted flow rate and pressure values. Observed–predicted plots for flow rate, P1, P2, and P3. In all plots, the horizontal axis shows predicted values from the equations, and the vertical axis shows the experimentally measured values. The gray dotted line indicates the Y = X line. P1; pre-pump pressure, P2; pre-oxygenator pressure, P3; post-oxygenator pressure

The overall R^2^, RMSD, bias (median and interquartile range), and median relative error (%) for flow rates were 0.96, 0.12, -0.13 (-0.16, -0.02), and -5.09%, respectively. The R^2^, RMSD, bias, and relative error (%) for each of the pressure measurements (P1, P2, and P3) were 0.97, 7.73, -3.96 (-8.62, -0.72), 7.05%, 0.96, 13.01, 6.27 (1.37, 15.82), 4.13%, and 0.96, 11.73, 3.12 (-1.29, 11.30), 2.97%, respectively. Table S3 (Additional File 1) lists the predicted and measured values for all combinations. Table S4 (Additional File 1) presents the R^2^, RMSD, and bias for flow rates and pressures (P1, P2, and P3). Figure 6 shows the predicted versus observed plots for flow rates and pressures (P1, P2, and P3). In all plots, the data points clustered around the Y = X line, demonstrating excellent predictive performance. The slope (and intercept) of the regression curves for flow rate, P1, P2, and P3 were 1.10614 (-0.38613), 1.08467 (-0.83229), 1.13631 (-16.17748), and 1.15533 (-14.89196), respectively. Detailed statistical analyses are provided in Appendix 3 (Additional File 1).

### Clinical cases

Overall, 12 sets of flow rate and circuit pressure (P1, P2, P3) data were obtained from three patients. The median (minimum to maximum) differences between the measured and predicted values (measured value minus predicted value) were 0.22 (–0.13 to 0.47) L/min, 2.38 (0.69 to 12.68) mmHg, -0.08 (–13.76 to 11.35) mmHg, and -1.31 (–8.67 to 5.63) mmHg, respectively.

## Discussion

### Clinical implications

This study was conducted to address the lack of standardized methods for predicting the optimal cannula size required to achieve appropriate ECMO flow and circuit pressures in veno-venous (V-V) ECMO. To this end, we developed a predictive formula for the ECMO flow rate and circuit pressures based on fluid dynamic principles and validated its accuracy using an experimental model. Specifically, the following three points were confirmed:

1) Increasing the diameter of the drainage or return cannula increased ECMO flow.

2) Higher pump speed increased flow rate, decreased P1, and increased P2 and P3.

3) Bed height did not affect ECMO flow rate but increased P1, P2, and P3.

Maintaining appropriate ECMO flow rate is essential to achieve adequate oxygenation. However, attaining higher flow rate typically requires larger-diameter cannulas, which may increase the risk of vascular injury. Therefore, understanding how different cannula combinations affect flow rate enables clinicians to select the optimal cannulas while balancing efficacy and safety.

Similarly, maintaining circuit pressures within a safe range is crucial for reliable ECMO management. For example, excessively negative P1 can cause serious problems. When P1 falls below the vapor pressure of the fluid, vapor bubbles may form, leading to cavitation [11]. Cavitation disrupts flow, reduces pump performance, and causes red blood cell damage and hemolysis, which may result in severe complications such as pulmonary embolism.

In this study, using the new four-step method, the ECMO flow rate and circuit pressures could be predicted with errors of only a few percent. Being able to predict the flow rate and circuit pressures before initiating ECMO is valuable not only for selecting appropriate cannulas, but also for the early detection of problems. For instance, a decrease in the flow rate accompanied by an increase in P1 and simultaneous decreases in P2 and P3 suggests pump malfunction. Conversely, a decrease in the flow rate with a uniform rise in P1, P2, and P3 indicates possible obstruction of the return cannula. Such abnormalities are usually detected through real-time monitoring. If ECMO begins without complications, these problems can be recognized by paying attention to subsequent “changes”. However, complications arising at the initiation of ECMO may be difficult to identify. Having predicted values in advance enables clinicians to detect such issues by comparing the “differences” between the predicted and measured values.

### Mechanistic explanations

Flow rate and circuit pressures interact dynamically rather than changing independently during ECMO operation. For instance, enlarging only the drainage cannula diameter reduces resistance in that segment and thus lowers pressure loss, which in turn increases ECMO flow rate. However, this higher flow rate raises the pressure loss across other components, such as the oxygenator and return cannula, thereby limiting the benefit of the larger drainage cannula. When comparing drainage 18 Fr/return 14 Fr with drainage 22 Fr/return 14 Fr, the latter configuration results in a smaller difference between central venous pressure (CVP) and P1, but greater differences between P2 and P3, and between P3 and CVP. Although such simultaneous changes can be confusing, they become clearer when analyzed step by step using the proposed four-step method.

Adjustments in pump speed and bed height influence P1 and provide a mechanistic rationale for bed height management to prevent cavitation. As pump speed increases to achieve higher ECMO flow, P1 becomes more negative. Raising bed height does not increase ECMO flow but elevates P1, P2, and P3, helping prevent cavitation. This effect arises from the hydrostatic pressure difference between the pump and bed levels. In other words, the pressure “borrowed” from the height difference between the bed and the pump to raise P1, P2, and P3 must later be “repaid” when the blood from the pump is pumped back to the bed level. This explains why the difference between P3 and CVP increases despite a constant ECMO flow, as shown in Fig. 5c. Using the present equation, the necessary bed height to prevent cavitation at specific pump speeds can be calculated in advance.

### Comparisons with existing methods

To date, various approaches have been explored to elucidate the effectiveness of ECMO from different perspectives. For instance, previous studies have attempted to estimate the ECMO flow rate using regression models derived from clinical data [4]. However, the vast number of possible combinations of drainage and return cannulas would require an unrealistically large dataset. In contrast, the fluid dynamics-based equation proposed in this study enables theoretical estimation without relying on empirical data fitting.

In addition, several previous studies have investigated the factors affecting the effectiveness of ECMO that were not incorporated into the present model. Oxygenation depends not only on the ECMO flow rate, but also on the recirculation, cardiac output, and circuit configuration [12, 13], with ECMO flow rate exerting a greater influence than cannula position [14]. Furthermore, studies examining the presence and location of side holes have provided important insights into flow reduction and recirculation under drainage-limited conditions [15–17].

### Educational value of the equation

Understanding ECMO using mathematical formulas based on fluid dynamics is also useful as a tool for systematically learning how ECMO works. For example, in clinical practice, the location of issues within an ECMO circuit can be inferred from variations in circuit pressures (Table S5 in Additional File 1). For trainees, interpreting these changes can be challenging and often depends on rote memorization. As illustrated in Fig. 5, this equation clarifies the hydrodynamic rationale underlying each pressure variation in P1, P2, and P3. For example, the collapse of the venous wall against the side holes of the drainage cannula is hydrodynamically equivalent to using a thinner cannula. By applying the four-step analytical method and systematically varying each parameter, trainees can develop a more comprehensive understanding of the physiological and mechanical principles governing ECMO circuit pressures. Consult Appendix 4 (Additional File 2) for illustrative examples of the method’s application.

### Limitations

This study has certain limitations. From the perspective of modeling assumptions, the effects of patient-specific vascular geometry and intravascular blood volume were not incorporated into the model. Consequently, in patients with drainage failure or other hemodynamic issues, the simulated results may not fully correspond to clinical measurements. For instance, in cases of drainage failure caused by hypovolemia, the side holes of the cannula may be tightly pressed against the vessel wall, preventing effective drainage from that region. A previous study used computational fluid dynamics to investigate how the number of side holes affects the flow characteristics of a cannula under specific pressure conditions [18]. By performing such simulations under various pressure conditions, it is possible to obtain a pressure–flow curve when the side holes are partially or completely occluded. Alternatively, pressure–flow curves can be measured experimentally by blocking a portion of the side holes. Incorporating these pressure–flow data into the model may enable simulation of clinical scenarios such as hypovolemia or partial side-hole obstruction.

From the perspective of experimental setup, it is worth noting that a glycerin solution is used instead of blood. The non-Newtonian properties of blood cannot be reproduced with a glycerin solution. Besides, in the absence of a catalog detailing the characteristics of the connecting tubes, simplified coefficients of the approximation curves were adopted. Because these coefficients affect the accuracy of the prediction formula, we selected reasonable values consistent with the Alfred ECMO guideline, which indicates that a pressure gradient of 100 mmHg across 1 m of 3/8-inch tubing results in approximately 5 L/min of blood flow [19].

## Conclusions

Our new four-step method is useful for predicting the ECMO flow rate and circuit pressure. This hydrodynamics-based predictive equation may enhance our understanding of ECMO and help prevent and detect clinical complications. In this study, clinical validation was performed in only three cases (Appendix 1, Additional File 1). Further validation of the ECMO flow and circuit pressures in actual clinical settings is warranted in future studies. Clinical data should preferably include detailed parameters, such as cannula size, pump speed, CVP, bed height, and hematocrit.

## Supplementary Information

Additional file1 (DOCX 2407 KB)

Additional file2 (XLSX 13 KB)

## Data Availability

All data generated or analyzed during this study are included in this published article and its supplementary information files**.**

## Abbreviations

ECMO: Extracorporeal membrane oxygenation
RMSD: Root mean square deviation
CVP: Central venous pressure
VV-ECMO: Venovenous ECMO
