# Supplementary material for: Simple and reliable method for predicting extracorporeal membrane oxygenation flow rates and circuit pressures
Source: Intensive Care Med Exp. 2026 Mar 4;14:28. doi: 10.1186/s40635-026-00870-z (PMC12960856; doi:10.1186/s40635-026-00870-z)
Supplement: Supplementary file 1 — Additional file1 (DOCX 2407 KB) [file 40635_2026_870_MOESM1_ESM.docx]

**Additional Files**

**Additional File 1**

**Appendix 1. Three clinical examples**

In all patients, the connecting tubes are the same as those used in the main text.

**Patient 1.** A 60-year-old female, height: 158 cm, weight: 64 kg

Patient undergoes cadaveric single-lung transplantation for idiopathic interstitial pneumonia.

Drainage cannula: HLS 21 Fr

Return cannula: Biomedicus 19 Fr

Bed height: 40 cm, central venous pressure (CVP) 10 mm Hg

Figure below shows the measured (black bar) and predicted (gray bar) extracorporeal membrane oxygenation (ECMO) flow rates at each pump speed (left), and the measured (black line) and predicted (gray line) values of pre-pump pressure (P1), pre-oxygenator pressure (P2), and post-oxygenator pressure (P3) at each pump speed (right).

**Patient 2.** A 33-year-old female (height: 153 cm; weight: 49 kg)

Patient undergoes bilateral cadaveric lung transplantation for bronchiectasis.

Drainage cannula: HLS 21 Fr

Return cannula: Biomedicus 17 Fr

Bed height: 40 cm; CVP: 10 mmHg

The figure below shows the measured (black bar) and predicted (gray bar) ECMO flow rates at each pump speed (left), and the measured (black line) and predicted (gray line) values of P1, P2, and P3 at each pump speed (right).

**Patient 3.** A 51-year-old male, (height: 180 cm; weight: 108 kg)

Patient undergoes tracheal stenting for airway stenosis.

Drainage cannula: PCKC V3 24 Fr

Return cannula: Biomedicus 19 Fr

Bed height: 30 cm; CVP: 10 mmHg

The figure below shows the measured (black bar) and predicted (gray bar) ECMO flow rates at each pump speed (left), and the measured (black line) and predicted (gray line) values of P1, P2, and P3 at each pump speed (right).

P ＝ (K1) * F^2 + (K2) * F + K3

| Component | | K1 | K2 | K3 |
| --- | --- | --- | --- | --- |
| Return cannula | Biomedicus 17 Fr | 8.2885 | 4.5481 | 0 |
|  | Biomedicus 19 Fr | 4.4863 | 3.2592 | 0 |
| Drainage cannula | HLS 21 Fr | 3.116 | 2.2771 | 0 |
|  | PCKC V3 24 Fr | 1.6464 | 1.8538 | 0 |

**Additional File 2**

**Table S1. Four steps to calculate the ECMO flow rate and circuit pressures**

| Step 1 | Create an approximate curve for each part of the ECMO circuit and the pump. |
| --- | --- |
| Step 2 | Find the intersection of the approximate curves of the circuit and the pump. |
| Step 3 | Calculate the pressure within the circuit starting from the drainage side. |
| Step 4 | Adjust for bed height. |

**Abbreviation**: ECMO: extracorporeal membrane oxygenation

**Additional File 3**

**Table S2. Coefficients of the return and drainage cannula approximation curves used in this study**

| Component | | K1 | K2 | K3 |
| --- | --- | --- | --- | --- |
| Return cannula | 14 Fr | 18.01 | 1.34 | 0 |
|  | 18 Fr | 5.01 | -1.74 | 0 |
| Drainage cannula | 18 Fr | 8.84 | 2.51 | 0 |
|  | 22 Fr | 3.09 | -0.54 | 0 |
| Oxygenator | | 0.55 | 15.33 | 0 |
| 3/8-inch connection parts | 1) Return cannula to pump | 3 | 3 | 0 |
|  | 2) Pump to oxygenator | 0.5 | 0.5 | 0 |
|  | 3) Oxygenator to return cannula | 3 | 3 | 0 |
| Pump | 2,500 rpm | -0.84 | -1.55 | 174 |
|  | 3,000 rpm | -1.03 | -0.48 | 248 |
|  | 3,500 rpm | -0.86 | -1.44 | 337 |

Coefficients for connection parts are applied by the authors.

Data are prepared based on the Senko Ika catalog.

Approximate curves: P ＝ (K1) * Q^2 + (K2) * Q + K3

P denotes pressure (mmHg), Q denotes flow rate (L/min), and K represents a coefficient specific to each cannula or pump.

**Additional File 4**

**Figure S1. Calculation of the pressure–flow curve for the entire circuit.** (a) Return cannula pressure–flow curve. (b) Oxygenator pressure–flow curve stacked on (a). (c) Drainage cannula pressure–flow curve stacked on (b). (d) Connection part 1 curve stacked on (c). (e) Connection part 2 curve stacked on (d). (f) Connection part 3 curve stacked on (e). In all graphs, the horizontal axis indicates the flow rate through the circuit, and the vertical axis shows the pressure drop.

**
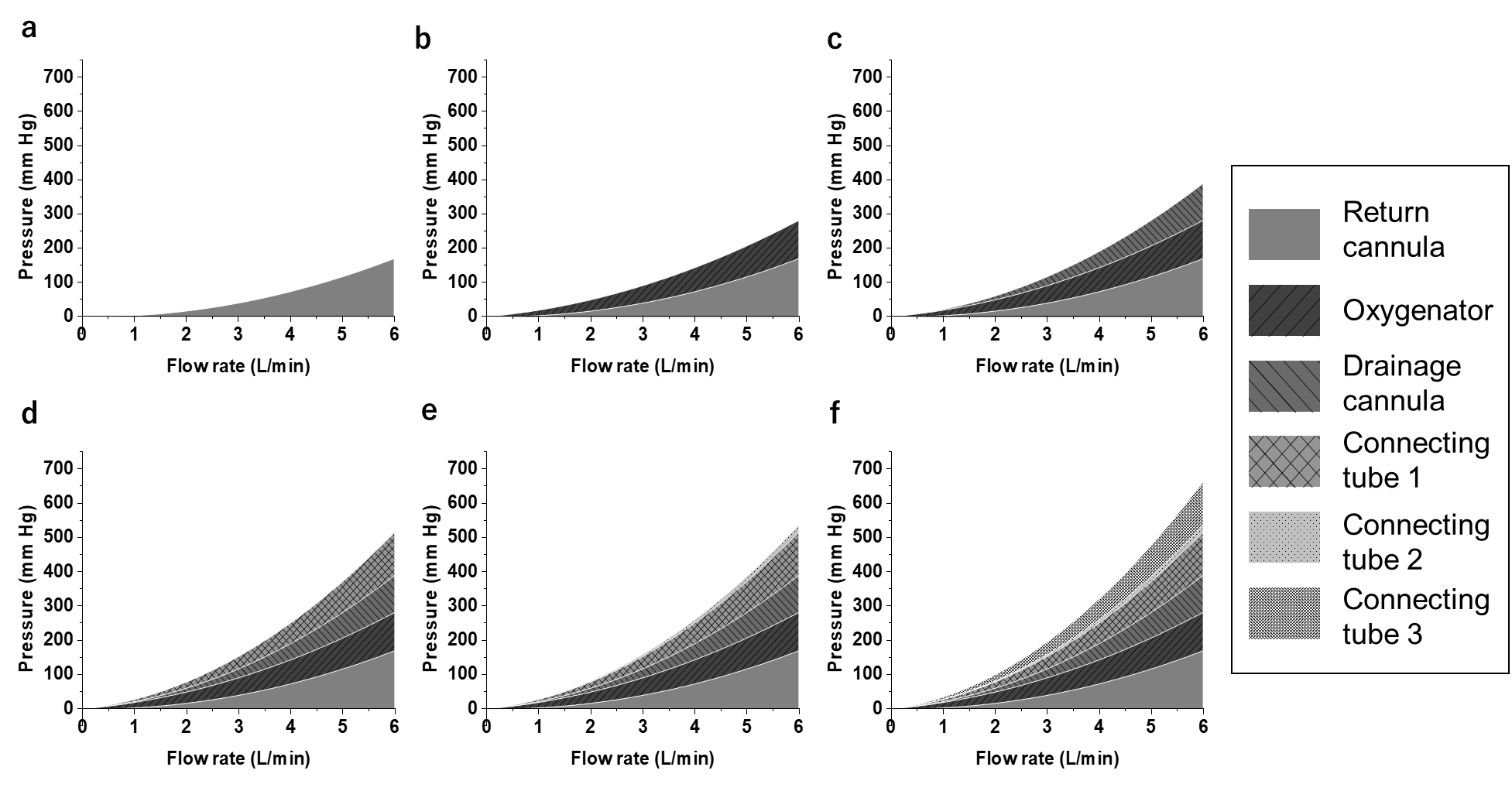
**

**Additional File 5**

**Appendix 2. Extended Bernoulli’s equation**

Bernoulli’s equation is a principle that demonstrates the law of conservation of energy in fluid flow. According to the equation, the sum of the following three types of energy remains constant: pressure energy ($p$), the energy due to the fluid pressure; kinetic energy ($\frac{1}{2}\rho v^{2}$), the energy from the fluid velocity; and potential energy ($\rho gz$), the energy due to the height of the fluid.

Expressed as an equation, it appears as follows:

$$p_{1}+\frac{1}{2}\rho{v_{1}}^{2}+\rho gz_{1}=p_{2}+\frac{1}{2}\rho{v_{2}}^{2}+\rho gz_{2}=const$$

where $p$ is the pressure; $\rho$, fluid density; $g$, acceleration due to gravity; $v$, flow velocity; $z$, height from a reference plane; and subscripts 1 and 2, 2 different points along a streamline.


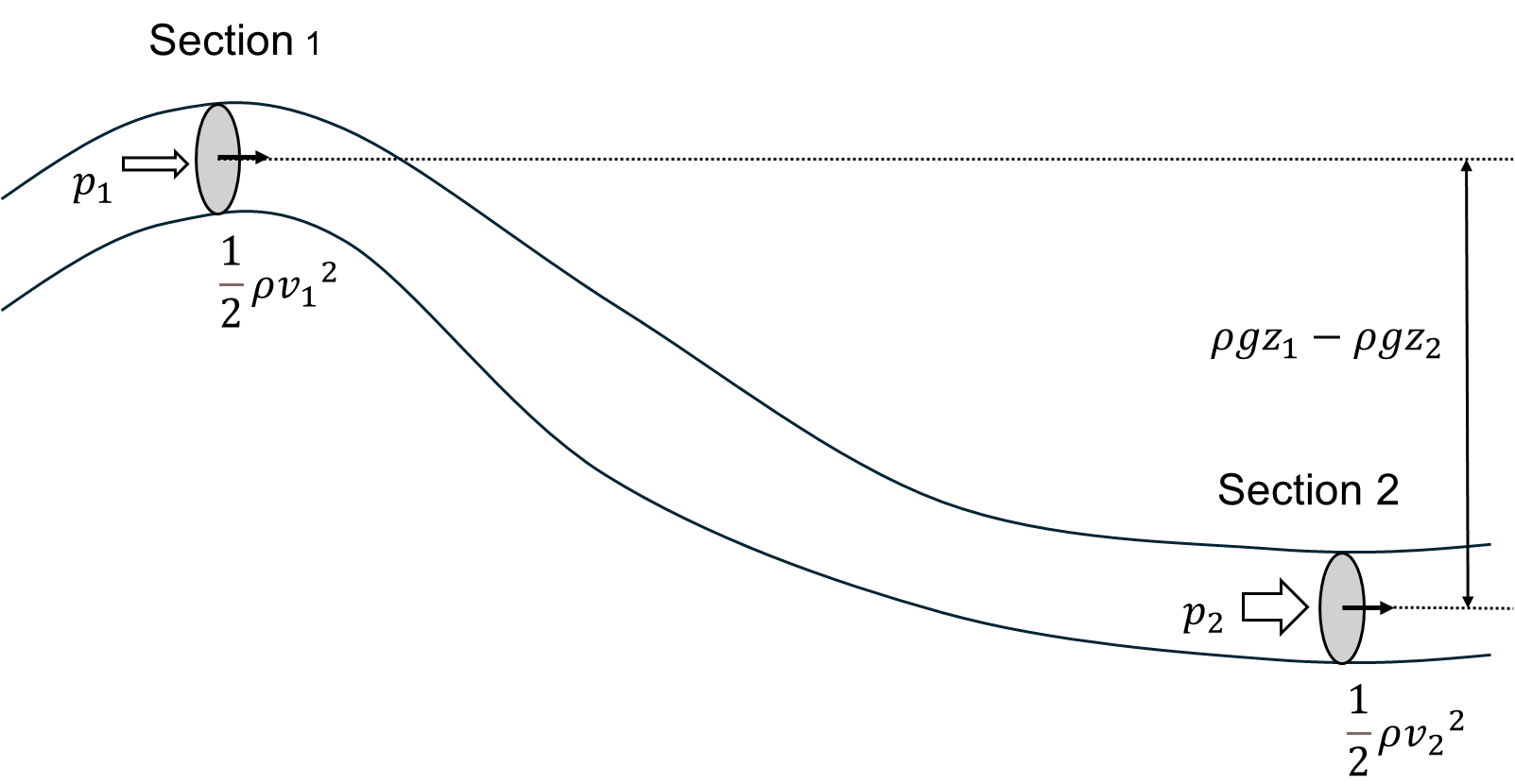


In this study, because both Sections 1 and 2 are connection tubes of 3/8 inch, the cross-sectional areas are equal. Thus, the flow velocities (= flow rate / cross-sectional area) are also equal. Therefore, the kinetic energies are also equal. If Section 1 is taken as the reference height, Section 2 will have lower potential energy than the reference. According to Bernoulli’s equation, this means that the pressure energy of section 2 is higher by that amount. However, in actual fluid flow, ideal conditions (no friction, no pumps or turbines) are rare. Therefore, to account for more realistic situations, the “extended Bernoulli’s equation,” is used. This equation takes the following elements into consideration:

Friction loss: The fluid loses energy due to friction with the pipe walls, bends, valves, etc. This lost energy is converted into heat.

Pump supply: The pump adds mechanical work to the fluid, increasing its energy.

Expressed as an equation, it appears as follows:

$$p_{1}+\frac{1}{2}\rho{v_{1}}^{2}+\rho gz_{1}+pump supply=p_{2}+\frac{1}{2}\rho{v_{2}}^{2}+\rho gz_{2}+friction loss$$

This corresponds to what is stated in the main text: “principles: (1) ECMO circuit components consume pressure; (2) the pump supplies pressure; and (3) the pressure consumed equals the pressure supplied.”

**Additional File 6**

**Figure S2. Adjustments for bed height.** Elevating the patient bed increases the circuit pressure in the lower sections, including the pump, connection tube 2, and oxygenator (P1, P2, and P3) due to the conversion of potential to pressure energy.

**Abbreviations:** P1; pre-pump pressure, P2; pre-oxygenator pressure, and P3; post-oxygenator pressure

**
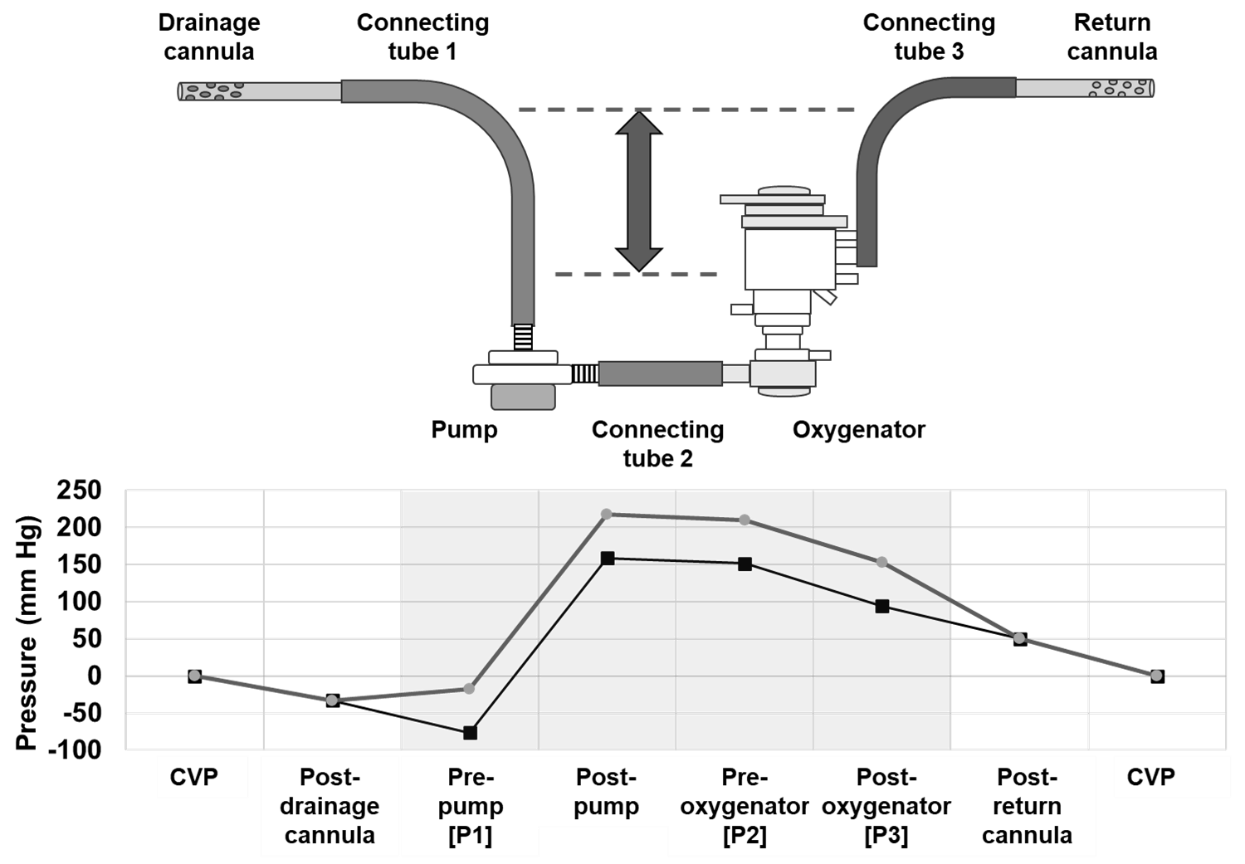
**

**Additional File 7**

**Appendix 3. Predictive performance of the four-step equation**

| ECMO parameters | Intercept | Slope | R^2^ |
| --- | --- | --- | --- |
| Flow rate | -0.38613 | 1.10614 | 0.99319 |
| P1 | -0.83229 | 1.08467 | 0.98934 |
| P2 | -16.17748 | 1.13631 | 0.99244 |
| P3 | -14.89196 | 1.15533 | 0.99166 |

The intercept and slope of each regression curve from the observed–predicted plots are presented. As the prediction accuracy is better when the intercept is close to 0 and the slope is close to 1, these results suggest that the prediction equation used in this study is adequate for clinical use.

The figure below shows effect of the combination of cannula size, pump speed, and bed height (horizontal axis) on the bias (experimental value minus calculated value) of the extracorporeal membrane oxygenation (ECMO) flow rate and circuit pressure (vertical axis).

We discuss the systematic errors noted in the observed–predicted plots. Nine points were observed for each combination of drainage and return cannulas. From left to right: 2500 rpm (bed heights of 0, 40, and 80 cm), 3000 rpm (bed heights of 0, 40, and 80 cm), and 3500 rpm (bed heights of 0, 40, and 80 cm). The ECMO flow rates tend to be overestimated with smaller inner cannula diameters. Regarding circuit pressure, when the return cannula was 14 Fr (smaller return cannula diameter), the calculated values of P2 and P3 were lower than the measured values. However, when both the return and drainage cannulas were 18 Fr (smaller drainage cannula diameter), the calculated value of P1 exceeded the measured value. These trends are more pronounced at higher pump speeds.

**
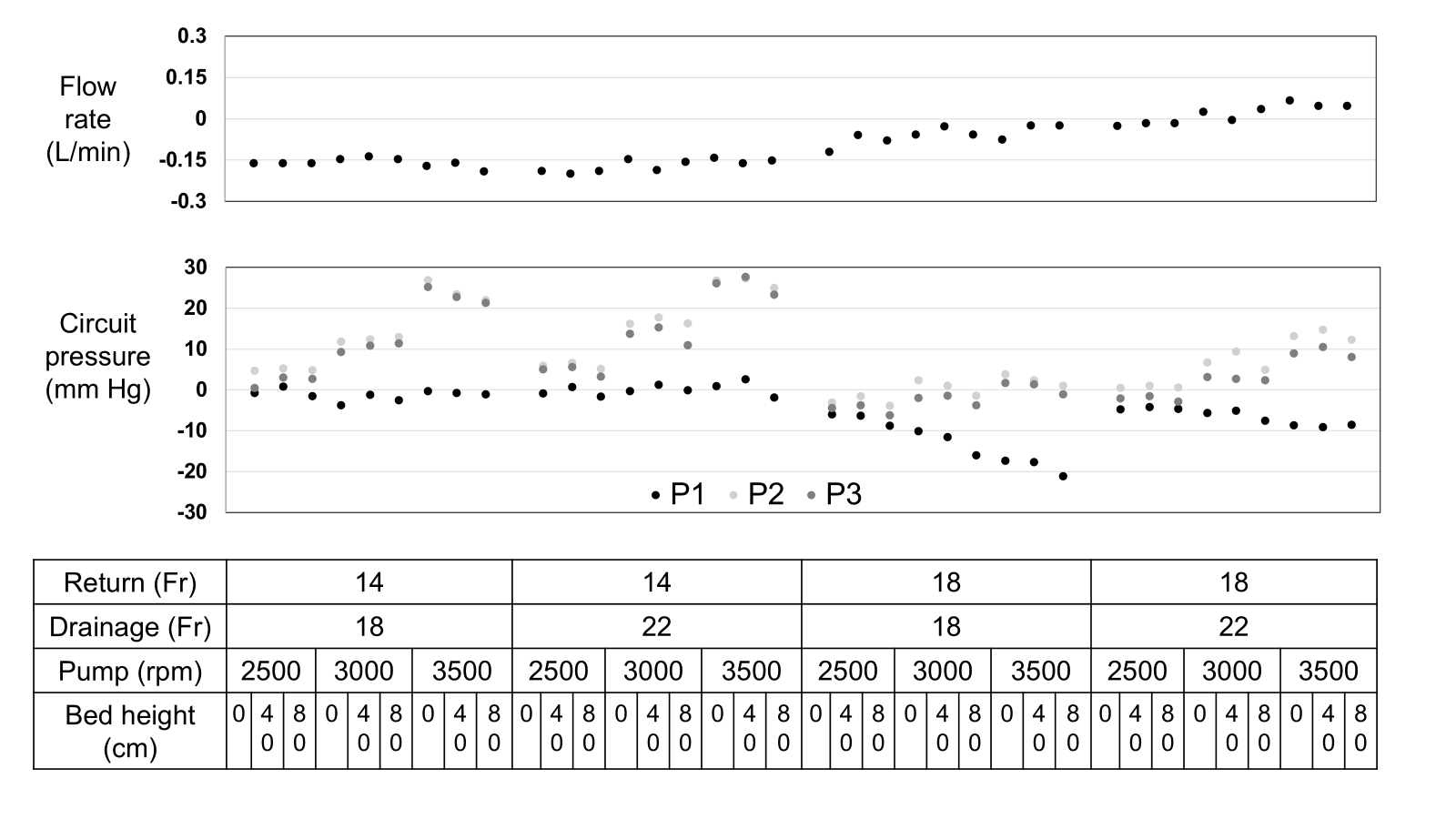
**

**Additional File 8**

**Table S3. Predicted and measured values for all combinations**

| Bed height | Return | Drainage | Pump speed | Flow  exp | P1  exp | P2  exp | P3  exp | Flow  cal | P1  cal | P2  cal | P3  cal |
| --- | --- | --- | --- | --- | --- | --- | --- | --- | --- | --- | --- |
| Cm | Fr | Fr | rpm | L/min | mm Hg | mm Hg | mm Hg | L/min | mm Hg | mm Hg | mm Hg |
| 0 | 14 | 18 | 2,500 | 1.72 | -53 | 118 | 83 | 1.88 | -52 | 113 | 83 |
| 40 | 14 | 18 | 2,500 | 1.72 | -22 | 148 | 115 | 1.88 | -23 | 143 | 112 |
| 80 | 14 | 18 | 2,500 | 1.72 | 5 | 177 | 144 | 1.88 | 7 | 172 | 141 |
| 0 | 14 | 18 | 3,000 | 2.17 | -80 | 173 | 132 | 2.32 | -76 | 161 | 123 |
| 40 | 14 | 18 | 3,000 | 2.18 | -48 | 203 | 163 | 2.32 | -47 | 191 | 152 |
| 80 | 14 | 18 | 3,000 | 2.17 | -20 | 233 | 193 | 2.32 | -17 | 220 | 182 |
| 0 | 14 | 18 | 3,500 | 2.58 | -105 | 244 | 196 | 2.75 | -105 | 217 | 171 |
| 40 | 14 | 18 | 3,500 | 2.59 | -76 | 270 | 223 | 2.75 | -75 | 247 | 200 |
| 80 | 14 | 18 | 3,500 | 2.56 | -47 | 298 | 251 | 2.75 | -46 | 276 | 230 |
| 0 | 14 | 22 | 2,500 | 1.88 | -32 | 139 | 104 | 2.07 | -31 | 133 | 99 |
| 40 | 14 | 22 | 2,500 | 1.87 | -1 | 169 | 134 | 2.07 | -2 | 162 | 128 |
| 80 | 14 | 22 | 2,500 | 1.88 | 26 | 197 | 161 | 2.07 | 28 | 192 | 158 |
| 0 | 14 | 22 | 3,000 | 2.4 | -46 | 206 | 161 | 2.55 | -46 | 190 | 147 |
| 40 | 14 | 22 | 3,000 | 2.36 | -15 | 237 | 192 | 2.55 | -16 | 219 | 177 |
| 80 | 14 | 22 | 3,000 | 2.39 | 13 | 265 | 217 | 2.55 | 13 | 249 | 206 |
| 0 | 14 | 22 | 3,500 | 2.88 | -62 | 283 | 231 | 3.02 | -63 | 256 | 205 |
| 40 | 14 | 22 | 3,500 | 2.86 | -31 | 313 | 262 | 3.02 | -34 | 286 | 234 |
| 80 | 14 | 22 | 3,500 | 2.87 | -6 | 340 | 287 | 3.02 | -4 | 315 | 264 |
| 0 | 18 | 18 | 2,500 | 2.21 | -83 | 82 | 42 | 2.33 | -77 | 85 | 46 |
| 40 | 18 | 18 | 2,500 | 2.27 | -54 | 113 | 72 | 2.33 | -48 | 114 | 76 |
| 80 | 18 | 18 | 2,500 | 2.25 | -27 | 140 | 99 | 2.33 | -18 | 144 | 105 |
| 0 | 18 | 18 | 3,000 | 2.82 | -124 | 121 | 68 | 2.88 | -114 | 119 | 70 |
| 40 | 18 | 18 | 3,000 | 2.85 | -96 | 149 | 98 | 2.88 | -84 | 148 | 99 |
| 80 | 18 | 18 | 3,000 | 2.82 | -71 | 176 | 125 | 2.88 | -55 | 177 | 129 |
| 0 | 18 | 18 | 3,500 | 3.35 | -175 | 161 | 100 | 3.42 | -158 | 157 | 98 |
| 40 | 18 | 18 | 3,500 | 3.4 | -146 | 189 | 129 | 3.42 | -128 | 187 | 128 |
| 80 | 18 | 18 | 3,500 | 3.4 | -120 | 217 | 156 | 3.42 | -99 | 216 | 157 |
| 0 | 18 | 22 | 2,500 | 2.68 | -56 | 108 | 60 | 2.71 | -51 | 108 | 62 |
| 40 | 18 | 22 | 2,500 | 2.69 | -26 | 138 | 90 | 2.71 | -22 | 137 | 91 |
| 80 | 18 | 22 | 2,500 | 2.69 | 3 | 167 | 118 | 2.71 | 8 | 166 | 121 |
| 0 | 18 | 22 | 3,000 | 3.37 | -82 | 158 | 97 | 3.34 | -76 | 151 | 94 |
| 40 | 18 | 22 | 3,000 | 3.34 | -52 | 190 | 126 | 3.34 | -47 | 181 | 123 |
| 80 | 18 | 22 | 3,000 | 3.38 | -25 | 215 | 155 | 3.34 | -17 | 210 | 153 |
| 0 | 18 | 22 | 3,500 | 4.05 | -115 | 215 | 141 | 3.98 | -106 | 202 | 132 |
| 40 | 18 | 22 | 3,500 | 4.03 | -86 | 246 | 172 | 3.98 | -77 | 231 | 162 |
| 80 | 18 | 22 | 3,500 | 4.03 | -56 | 273 | 199 | 3.98 | -48 | 261 | 191 |

**Additional File 9**

**Table S4. R^2^, RMSD, and bias of flow rates P1, P2, and P3**

| ECMO parameters | R^2^ | RMSD | Bias (median and interquartile range) |
| --- | --- | --- | --- |
| Flow rate | 0.96 | 0.12 | -0.13 (-0.16, -0.02) |
| P1 | 0.97 | 7.73 | -3.96 (-8.62, -0.72) |
| P2 | 0.96 | 13.01 | 6.27 (1.37, 15.82) |
| P3 | 0.96 | 11.73 | 3.12 (-1.29, 11.30) |

**Abbreviations**: RMSD: root mean square deviation; P1: pre-pump pressure; P2: pre-oxygenator pressure; P3: post-oxygenator pressure

**Additional File 10**

**Table S5. Circuit pressure changes caused by error**

| Location of issues | Flow | P1 | P2 | P3 |
| --- | --- | --- | --- | --- |
| Drainage error | Down | Down | Down | Down |
| Pump failure | Down | UP | Down | Down |
| Oxygenator coagulation | Down | UP | UP | Down |
| Return error | Down | UP | UP | UP |
